# Availability and use of technology for e-learning: to what extent do these impact Bangladeshi university students? A cross-sectional study

**DOI:** 10.12688/f1000research.75532.1

**Published:** 2021-12-15

**Authors:** Md. Kamrul Hasan, Tajrin Tahrin Tonmon, Humayun Kabir, Sumaya Binte Masud, Md. Abeed Hasan, Bikash Das, Monira Akter, Mohammad Delwer Hossain Hawlader, Dipak Kumar Mitra

**Affiliations:** 1 Department of Public Health, North South University, Dhaka, 1229, Bangladesh; 2 IQARUS, Cox’s Bazar, 4700, Bangladesh; 3 Obstetrical and Gynaecological Society of Bangladesh, Dhaka, 1207, Bangladesh; 4 International Organization for Migration (IOM), Cox's Bazar, Bangladesh; 5 Institute of Social Welfare & Research, University of Dhaka, Dhaka, 1000, Bangladesh

**Keywords:** E-learning, availability of technology, use of technology, readiness, stress

## Abstract

**Background: **E-learning is making education globally and conveniently attainable with the deliverance of advanced technology. However, this mode of academia is still not commonly practiced locally. Thus, the study aimed to investigate technological availability, usability, and association to university students' perceived stress due to e-learning curriculum.

**Methods: **A cross-sectional study commenced among Bangladeshi university students enrolled in the e-learning curriculum. A total of 1162 university students were included. The main explanatory variables were related to the availability of technology and the use of technology. The outcome variable was perceived e-learning stress. In statistical analysis, *p*-value < 0.05 was considered statistically significant with a 95% confidence interval.

**Results: **In this study, lack of technological availability and usability were associated with higher level of perceived e-learning stress. Being female, living in rural areas, and outside of Dhaka division were found the associated factors in the lack of technological availability and usability.

**Conclusions: **A significant association between the availability and usability of technology with perceived e-learning stress was observed. Thus, measures should be taken to initialize e-learning adaptivity by increasing technological growth across the nation, considering educational preparedness for future catastrophes.

## 1. Introduction

With the widespread of coronavirus disease (COVID-19), measures are being taken to control the spreading while not hampering the success of academia. Educators and their students have encountered the e-learning process globally ( Bao, 2020). The vast gain in the influence of technology since the 1990s has been impacting the education system, amongst which e-learning is being adopted globally ( Barrot, Llenares, & del Rosario, 2021). Accordingly, the ‘digitalization’ process in education has been launched attached with a series of virtual management and digital competencies ( Purnama, Ulfah, Machali, Wibowo, & Narmaditya, 2021). Keeping pace with that, the effectiveness of e-learning is dependent on the optimism towards it and having fluency in technologies ( Çevik & Bakioğlu, 2021). Computer competency, especially internet usability, has powered a prodigious growth in e-learning, where institutions have integrated virtual classrooms into their curriculum to continue education ( Almossa, 2021; Mgutshini, 2013). This adoption has provided opportunities for students to enhance their competencies ( Churiyah, Sholikhan, Filianti, & Sakdiyyah, 2020; Siron, Wibowo, & Narmaditya, 2020) and attributed it to a requirement of developing digital literacy skills ( Phuapan, Viriyavejakul, & Pimdee, 2016; Purnama *et al.*, 2021).

Recently, various web-based free accessed platforms have been used to continue education ( Ali, Ramay, & Shahzad, 2011; Keskin & Yurdugül, 2020; Salta, Paschalidou, Tsetseri, & Koulougliotis, 2021). This virtual learning-enabled communication is convenient, especially for introverts ( Salta *et al.*, 2021; Walker, Rossi, Anastasi, Gray-Ganter, & Tennent, 2016). Nevertheless, obstacles remain in technological dilemmas, poor internet providers and limited adaptability, creating hurdles for the students ( Al-Amin *et al.*, 2021; Katz, Jordan, & Ognyanova, 2021; Shaukat, Niazi, Qazi, & Basit, 2021). Studies have shown that 70% of online students have faced technological errors or computer issues in this web-based pedagogical system ( Al-Amin *et al.*, 2021; Yan *et al.*, 2021). Hence, users’ self-ability in e-learning was noted to affect the learning experience ( Prifti, 2020; Sarwar *et al.*, 2020). Alqurashi (2016) investigated computer and internet usability and showed a satisfactory experience was positively and significantly associated with e-learning exposure in several studies ( Alqurashi, 2016; Daniels, Goegan, & Parker, 2021; Liu & Larose, 2008; Saif Almuraqab, 2020).

Adequate technological support was needed for the students to explore lesson materials. Several research assessed that students were getting anxious while acquiring lessons due to technological issues ( Ali *et al.*, 2011; Alzahrani & Seth, 2021; Çevik & Bakioğlu, 2021; Maheshwari, 2021). Whereas, with adequate technological support, students retrieved classes from anywhere at any time ( Al-Azzam, Elsalem, & Gombedza, 2020; Kumar, Kumar, & Ting, 2021). The e-learning experience in this context varied on factors like gender, residence, region and minority groups; ( Abuhassna *et al.*, 2020; Alqurashi, 2019; Alyoussef, 2021; Shahzad, Hassan, Aremu, Hussain, & Lodhi, 2021). Hence, increasing the dropout rates ( Cromley & Kunze, 2021; Yang, Peng, Wong, & Chong, 2021).

Besides, the transition from traditional to e-learning has created numerous issues for educators and university students of various disciplines ( Giray, 2021; Hsu, Wang, & Levesque-Bristol, 2019). As the curriculum was not designed for online learning ( Dong *et al.*, 2020; Misirli & Ergulec, 2021), it was reported as complicated to bring a change into it. Additionally, unequipped digital resources accelerated the complication for the learners in remote areas ( Rahiem, 2021). Over time, unequal access and distribution of adequate resources have been established due to differences in backgrounds ( Yu, Zhang, & Zou, 2021). Again, the various home environments of the students do not allow an equal environment for all, which is opposed by the traditional education environment ( Kumar *et al.*, 2021). Therefore, the call for equity is a matter of concern, and the issues of technological inequalities and digital disparities are foremost to be disrupted ( Klümper *et al.*, 2016). The United Nations Educational, Scientific and Cultural Organization-International Institute for Educational Planning has already emphasized that maintaining equity is a great challenge with the transition to learning remotely ( Istenič, 2021).

When researched further, perceived stress of e-learning was also found as one of the outcomes of the e-learning curriculum ( Alqurashi, 2016). With the deadly COVID-19 condition, young students are victims of accelerated stress, anxiety, and depression development, hindering their growth in education ( Shaukat *et al.*, 2021; Vayre & Vonthron, 2019). Studies have shown that e-learning during this pandemic has created psychological and intellectual gaps among students because of resource constraints ( Al-Balas *et al.*, 2020; Yang, Quadir, Chen, & Miao, 2016; Yang *et al.*, 2021). Students worldwide were already worried about the impact of the virus ( Wang & Zhao, 2020; Wang *et al.*, 2020), and the pedagogical changes found increased stress level among females particularly ( Almossa, 2021).

According to the United Nations, the disruption due to COVID-19 in the education sector impacted more than 72% of the students in the world ( Kumar *et al.*, 2021; Sarwar *et al.*, 2020). To overcome the disruption, internet, audio and video lectures, television and radio broadcasts, and available CDs have been used as lessons for the students in Pakistan. Kapasia *et al*. (2020) examined in India that students who belonged to remote regions and underprivileged residences were confronted with numerous issues related to mental health, network connectivity, and unsupportive home conditions ( Kapasia *et al.*, 2020). Meanwhile, Indonesian students also complained about technological interference, costly internet, lack of digital skills, and difficulties accessing learning materials ( Rahiem, 2020).

Meanwhile, in Bangladesh, universities started conducting online education in accordance with the declaration of the Ministry of Education. However, studies found disinterest among the students due to unpreparedness and lack of proper technological services ( Emon, Alif, & Islam, 2020). In contrast, a study found that 70% of the attendees of online classes belonged to private universities ( Ramij & Sultana, 2020) ( Islam *et al.*, 2020). The Bangladeshi university students are willing to learn online, although the affordability of technological tools' has hindered their willingness to continue ( Al-Amin *et al.*, 2021). Though online classes are not new for neighboring countries, keeping up with online classes extensively in developing countries like Bangladesh is not free of problems ( Shaukat *et al.*, 2021). However, sufficient studies have not been conducted nationwide scrutinizing these issues so far.

Therefore, this study aimed to observe the prevalence of technological availability and diversity of using technology to derive usability among university students. In addition, the study investigated students’ technological availability and usability association to perceived stress due to e-learning. This study may help policymakers, aiding them in coming up with unique solutions to improve the quality and equity of education.

## 2. Methodology

### 2.1 Study design, population, and setting

In Bangladesh, a cross-sectional study was conducted between December 26, 2020, and January 11, 2021. The inclusion criteria included: (a) enrolled Bangladeshi university students, (b) age at least 18 years, (c) enrolled e-learning curriculum for 30 days during the pandemic, and (d) participated by giving online consent.

### 2.2 Data collection procedure

Due to the COVID-19 pandemic, face-to-face data collection was prohibited. Therefore, data for this study were collected online (using “Google Forms”), followed by convenience and snowball sampling methods when schools were closed due to COVID-19 pandemic. The questionnaire link was posted in university students' social media groups (Facebook, Messenger, and WhatsApp). After screening all collected data and dropping data with missing responses, 1162 completed responses were included in the final analysis.

### 2.3 Availability and use of technology measures

The study’s explanatory variables were e-learning related information such as (a) availability of technology (6 items) included hardware and software facilities, internet speed and stability, computer access and internet connectivity for the technology availability subdomain, and (b) use of technology (11 items) included information via the internet, e-mail communication, office software, programming software, messaging apps, web tools, file hosting services, learning management systems, online forums, internet connectivity via mobile technologies. This measure was adopted by an e-learning readiness questionnaire ( Kabir *et al.*, 2021; Soydal, Alir, & Ünal, 2011; Ünal, Alir, & Soydal, 2014). The responses were in a five-point Likert scale of 1 for “strongly disagree,” and 5 for “strongly agree.” The availability of technology score ranges from 6 to 30, and the score of use of technology ranges from 11 to 55. However, the responses can be categorized into (1) sub-optimum and (2) optimum. Identification of optimum response was defined as the mean score of the item is 3.40 or greater ( Akaslan & Law, 2011; Soydal *et al.*, 2011; Ünal *et al.*, 2014).

### 2.4 Perceived e-learning stress measures

The outcome of e-learning, perceived e-learning stress was measured using a self-reported 14 items’ perceived stress scale (PSS). The total scores can range from 0 to 56. However, the PSS is restricted to measure perceived stress in the last month. We used PSS to measure the e-learning related stress by making a slight phrasal modification ( Lazarevic & Bentz, 2020; Program, 1983). Without changing the actual meaning of PSS, the modification was performed by adding the term “of e-learning” with each item to adjust the context to e-learning by following Lazarevic *et al*. (Cronbach’s alpha = 0.83) ( Lazarevic & Bentz, 2020). In PSS, an item “In the last month, how often have you found that you could not cope with all the things that you had to do?” After the modification, it looked like, “In the last month of e-learning, how often have you found that you could not cope with all the things that you had to do?” The response is in a five-point Likert scale of 0 for “Never” and 4 for “Very often”. To calculate total scores, reversed the scores (e.g., 0 = 4, 1 = 3 … & 4 = 0) of four positively response items (items 4, 5, 7, & 8) and then have to sum all the 14 items. For PSS, Cronbach’s alpha was found 0.86, which indicated good internal consistency.

### 2.5 Statistical analysis

All analyses were carried out using statistical software packages Stata version 16 (StataCorp, Texas, US) (RRID: SCR 012763). Initially, descriptive and inferential statistics such as central tendency, chi-squared (χ ^2^) tests, and t-test were carried out. The chi-square test was performed to examine the associations of technology availability categories and technology usability categories with different sample characteristics by gender, residence, and division. The association of availability and usability of technology categories with perceived stress score was examined using the t-test. Subsequently, the Pearson correlation coefficient test was used for examining the correlation of the sum score of availability and use of technology and PSS score. All statistical tests were two-sided, and a *p*-value < 0.05 was considered statistically significant for all the tests performed at 95% confidence interval.

### 2.6 Ethical considerations

All the procedures were conducted following the institution's review board (IRB) of North South University, Bangladesh (Memo no. 2021/OR-NSU/IRB/0601). The ethical standards laid down in the 1964 Declaration of Helsinki and its later amendments or comparable ethical standards were followed wherever applicable. Electronic consent was attached for all the participants involved in the study, and the respondents who were willing to participate were only considered as respondents. However, the study's nature, purpose, and objective were clearly stated, along with the declaration of confidentiality and anonymity on the first page of the questionnaire.

## 3. Results

### 3.1 Distribution of availability of technology by gender, residence, and division

The distribution of technological availability related variables are presented by gender, residence, and division are presented in Table 1. Compared to males, females were at more sub-optimum level with hardware facilities (55.64%; *p* = 0.004), software facilities (57.34%; *p* < 0.001), access to a computer whenever needed (56.83%; *p* = 0.001) and internet connectivity whenever needed (55.66%; *p* = 0.01). The rural students were at a sub-optimum level with hardware facilities (41.04%; *p* = 0.004), software facilities (41.60%; *p* = 0.002), good internet speed (41.94%; *p* < 0.001), satisfactory internet stability (41.76%; *p* < 0.001), access to a computer whenever needed (46.25%; *p* < 0.001) and internet connectivity whenever needed (45.61%; *p* < 0.001) compared to urban students. Along with this, the students outside the Dhaka division were at more non-optimized hardware facilities (32.95%; *p* = 0.002), non-optimized software facilities (33.74%; *p* < 0.001), more sub-optimal access to high internet speeds (33.42%; *p* < 0.001), higher levels of sub-optimal satisfaction with stability of the internet connection (33.38%; *p* < 0.001), higher levels of sub-optimal access to a computer whenever needed (36.69%; *p* < 0.001) and more sub-optimum connectivity to the internet whenever needed (35.89%; *p* < 0.001) compared to the students of Dhaka division.

**Table 1.** Distribution of availability of technology by gender, residence and division and association with perceived e-learning stress.

Availability of technology	Gender		*p*-value	Residence		*p*-value	Division		*p*-value	Stress score		*p*-value
	Male (n; %)	Female (n; %)		Urban (n; %)	Rural (n; %)		Dhaka (n, %)	Out of Dhaka (n; %)		Mean	t	
**The hardware facilities are enough**												
Suboptimum	307; 44.36%	385; 55.64%	**0.004**	408; 58.96%	284; 41.04%	**0.004**	464; 67.05%	228; 32.95%	**0.002**	30.02	6.07	**<0.001**
Optimum	249; 52.98%	221; 47.02%		316; 67.23%	154; 32.77%		354; 75.32%	116; 24.68%		27.91		
**The software facilities are enough**												
Suboptimum	282; 42.66%	379; 57.34%	**<0.001**	386; 58.40%	275; 41.60%	**0.002**	438; 66.26%	223; 33.74%	**<0.001**	30.24	7.27	**<0.001**
Optimum	274; 54.69%	227; 45.31%		338; 67.47%	163; 32.53%		380; 75.85%	121; 24.15%		27.76		
**The speed of the internet access is satisfactory**												
Suboptimum	361; 46.58%	414; 53.42%	0.221	450; 58.06%	325; 41.94%	**<0.001**	516; 66.58%	259; 33.42%	**<0.001**	30.08	7.61	**<0.001**
Optimum	195; 50.39%	192; 49.61%		274; 70.80%	113; 29.20%		302; 78.04%	85; 21.96%		27.35		
**The stability of the internet access is satisfactory**												
Suboptimum	349; 46.41%	403; 53.59%	0.184	438; 58.24%	314; 41.76%	**<0.001**	501; 66.62%	251; 33.38%	**<0.001**	30.09	7.39	**<0.001**
Optimum	207; 50.49%	203; 49.51%		286; 69.76%	124; 30.24%		317; 77.32%	93; 22.68%		27.48		
**I have access to computer whenever I need**												
Suboptimum	253; 43.17%	333; 56.83%	**0.001**	315; 53.75%	271; 46.25%	**<0.001**	371; 63.31%	215; 36.69%	**<0.001**	30.20	6.13	**<0.001**
Optimum	303; 52.60%	273; 47.40%		409; 71.01%	167; 28.99%		447; 77.60%	129; 22.405		28.12		
**I can connect internet whenever I need**												
Suboptimum	278; 44.34%	349; 55.66%	**0.010**	341; 54.39%	286; 45.61%	**<0.001**	402; 64.11%	225; 35.89%	**<0.001**	30.44	8.18	**<0.001**
Optimum	278; 51.96%	257; 48.04%		383; 71.59%	152; 28.41		416; 77.76%	119; 22.24%		27.68		

### 3.2 Distribution of use of technology by gender, residence, and division

In Table 2, distribution of technological usability related variables by gender, residence, and division are presented. The females were at more sub-optimum level of office software usability (59.72%, *p* < 0.001), programming language software usability (55.06%, *p* = 0.001), web 2.0 tools usability (55.19%, *p* < 0.001), file hosting services usability (56.17%, *p* = 0.005) and learning management systems usability (55.03%, *p* < 0.001). Alongside, the rural students were at more sub-optimum level of internet usability (45.14%, *p* < 0.001), e-mail communication (42.56%, *p* = 0.001), office software usability (46.64%, *p* < 0.001), social network usability (45.69%, *p* = 0.01), instant messaging usability (42.58%, *p* < 0.001), Web 2.0 tools usability (39.57%, *p* = 0.02), file hosting services usability (44.00%, *p* < 0.001) and learning management systems usability (39.26%, *p* = 0.04). Suboptimum usability of technology was found to be higher among the students outside the Dhaka division for internet usability (38.52%, *p* < 0.001), e-mail communication (34.33%, *p* = 0.001), office software usability (34.98%, *p* < 0.001), programming language software usability (31.76%, *p* = 0.008), social network usability (36.04%, *p* = 0.03), instant messaging usability (32.14%, *p* = 0.046), file hosting services usability (34.50%, *p* < 0.001), learning management systems usability (31.54%, *p* = 0.008), online forum usability (33.86%, *p* = 0.002) and mobile technologies usability (40.48%, *p* < 0.001).

**Table 2.** Distribution of use of technology by gender, residence and division and association with perceived e-learning stress.

Use of technology	Gender		*p*-value	Residence		*p*-value	Division		*p*-value	Stress score		*p*-value
	Male (n; %)	Female (n; %)		Urban (n; %)	Rural (n; %)		Dhaka (n, %)	Out of Dhaka (n; %)		Mean	t	
**I use internet as information source**												
Suboptimum	128; 49.81%	129; 50.19%	0.477	141; 54.86%	116; 45.14%	**<0.001**	158; 61.48%	99; 38.52%	**<0.001**	30.42	3.89	**<0.001**
Optimum	428; 47.29%	477; 52.71%		583; 64.42%	322; 35.58%		660; 72.93%	245; 27.07%		28.81		
**I use e-mail as the main communication tool with my teachers and classmates**												
Suboptimum	271; 47.46%	300; 52.54%	0.795	328; 57.44%	243; 42.56%	**0.001**	375; 65.67%	196; 34.33%	**0.001**	29.80	3.60	**<0.001**
Optimum	285; 48.22%	306; 51.78%		396; 67.01%	195; 32.99		443; 74.96%	148; 25.04%		28.56		
**I use office software (e.g. M.S. PowerPoint, Word, Excel)**												
Suboptimum	228; 40.28%	338; 59.72%	**<0.001**	302; 53.36%	264; 46.64%	**<0.001**	368; 65.02%	198; 34.98%	**<0.001**	29.75	3.28	**<0.001**
Optimum	328; 55.03%	268; 44.97%		422; 70.81%	174; 29.1%		450; 75.50%	146; 24.50%		28.62		
**I use social network sites (e.g. Facebook, Twitter)**												
Suboptimum	96; 48.73%	101; 51.27%	0.786	107; 54.31%	90; 45.69%	**0.011**	126; 63.96%	71; 36.04%	**0.030**	29.81	1.69	0.092
Optimum	460; 47.67%	505; 52.33%		617; 63.94%	348; 36.06%		692; 71.71%	273; 28.29%		29.04		
**I use specific software (e.g. SPSS)**												
Suboptimum	382; 44.94%	468; 55.06%	**0.001**	516; 60.71%	334; 39.29%	0.063	580; 68.24%	270; 31.76%	**0.008**	29.19	0.19	0.848
Optimum	174; 55.77%	138; 44.23%		208; 66.67%	104; 33.33%		238; 76.28%	74; 23.72		29.11		
**I use instant messaging (e.g. Google Talk, Skype)**												
Suboptimum	281; 45.84%	332; 54.16%	0.148	352; 57.42%	261; 42.58%	**<0.001**	416; 67.86%	197; 32.14%	**0.046**	29.65	2.99	**0.003**
Optimum	275; 50.09%	274; 49.91%		372; 67.76%	177; 32.24%		402; 73.22%	147; 26.78%		28.62		
**I use Web 2.0 tools (e.g. Blog, wiki) to share information**												
Suboptimum	393; 44.81%	484; 55.19%	**<0.001**	530;60.43%	347; 39.57%	**0.021**	602; 68.64%	275; 31.36%		29.32	1.56	0.120
Optimum	163; 57.19%	122; 42.81%		194; 68.07%	91; 31.93%		216; 75.79%	69; 24.21%		28.70		
**I use file hosting services (e.g. Google Documents, Dropbox)**												
Suboptimum	263; 43.83%	337; 56.17%	**0.005**	336; 56.00%	264; 44.00%	**<0.001**	393; 65.50%	207; 34.50%	**<0.001**	29.71	3.26	**<0.001**
Optimum	293; 52.14%	269; 47.86%		388; 69.04%	174; 30.96%		425; 75.62%	137; 24.385		28.59		
**I use learning management systems (e.g. Blackboard, Moodle)**												
Suboptimum	402; 44.97%	492; 55.03%	**<0.001**	543; 60.74%	351; 39.26%	**0.044**	612; 68.46%	282; 31.54%	**0.008**	29.45	3.00	**0.003**
Optimum	154; 57.46%	114; 42.54%		181; 67.54%	87; 32.46%		206; 76.87%	62; 23.13%		28.22		
**I use online forums and chat to communicate with my colleagues**												
Suboptimum	263; 45.90%	310; 54.10%	0.189	347; 60.56%	226; 39.44%	0.225	379; 66.14%	194; 33.86%	**0.002**	29.61	2.53	**0.012**
Optimum	293; 49.75%	296; 50.25%		377; 64.01%	212; 35.99%		439; 74.53%	150; 25.47%		28.74		
**I use mobile technologies (e.g. Smartphone, Tablet) to connect internet**												
Suboptimum	107; 50.95%	103; 49.05%	0.320	120; 57.14%	90; 42.86%	0.088	125; 59.52%	85; 40.48%	**<0.001**	29.30	0.36	0.718
Optimum	449; 47.16%	503; 52.84%		604; 63.45%	348; 36.55%		693; 72.79%	259; 27.21%		29.17		

### 3.3 Association between the availability of technology and perceived e-learning stress

The association between the availability of technology and perceived e-learning stress is presented in Table 1. The mean scores of e-learning stress was significantly higher for a sub-optimum level of hardware facilities ( *p* < 0.001), software facilities ( *p* < 0.001), internet speed ( *p* < 0.001), internet stability ( *p* < 0.001), access to computer when needed ( *p* < 0.001), and connectivity to internet whenever needed ( *p* < 0.001).

### 3.4 Association between use of technology and perceived e-learning stress

Association between use of technology and perceived e-learning stress are presented in Table 2. The higher mean scores of e-learning stress were observed among the sub-optimum categories for internet usability ( *p* < 0.001), e-mail communication ( *p* < 0.001), office software usability ( *p* < 0.001), instant messaging usability ( *p* = 0.003), file hosting services usability ( *p* < 0.001), learning management systems ( *p* = 0.003), and online forum usability ( *p* = 0.012).

### 3.5 Correlation between the availability of technology, use of technology & PSS

The score of availability of technology, use of technology, and PSS was significantly correlated ( Table 3). The result showed that the availability of technology was positively correlated with the technology ( *p* < 0.001). On the other hand, the availability of technology was negatively correlated with the PSS score ( *p* < 0.001).

**Table 3.** Correlation between the scores of availability of technology, use of technology & perceived stress scale (PSS).

Availability of technology score	r - value	*p* - value
Use of technology score	0.65	**<0.001**
PSS score	−0.30	**<0.001**

## 4. Discussion

This study investigated the prevalence of technology availability and usability among university students. All the factors defining e-learning readiness in the context of technological availability and usability are significantly associated with perceived e-learning stress. To the best of the authors' knowledge, no other studies in developing countries like Bangladesh accessed the prevalence of students' technological availability and uasability.

In this study, sub-optimum hardware and software facilities were found to be significantly associated with the students' perceived stress score. The prevalence of these facilities was sub-optimum, especially among females, rural students, and students outside Dhaka. In an Indonesian study, Churiyah *et al*. (2020), observed that the lack of electronic devices and technological infrastructure in institutions had hampered the students' educational growth. Conversely, Alghamdi *et al*. (2020), identified that American students had performed better in online learning structure than the traditional settings with the help of necessary technological support, e.g., video and audio lectures, accessible communication etc. From our country’s perspective, the availability of hardware and software devices is comparatively prominent in Dhaka. Students residing in rural settings tend to be less facilitated regarding technical support because of financial and lack of digitalization contexts. Bangladesh is being digitalized adequately recently, which is also available within a homogenous stratum, e.g., citizens of Dhaka. As availability concerns were being addressed, the priority put the males in front of the queue, cooperating with the findings of our study. With the concerning availability of device facilities, stress is a common outcome for university students whose education is still entirely dependent on e-learning.

It was also found that on dissatisfactory level, sub-optimal internet speed and internet stability were two other factors found associated with females, rurality, and residency outside Dhaka when stratified. It, too, had a significant effect on the students’ stress levels. Barrot *et al*. (2021), stated that among the Philippine's students, the technological barrier at home, poor internet connectivity, and stability hindered their education and caused a significant challenge. In Bangladesh, internet speed and stability rely on the connection providers, and due to the sudden pandemic situation and lockdown at short notice, the internet usage and human dependency on it raised, which might have increased the pressure for the internet connection providers. Ali *et al*. (2011), in Pakistan, and Almossa *et al*. (2021) in Saudi Arabia showed students’ dissatisfaction due to issues with the internet. Moreover, it might be a profound reason for students’ e-learning stress.

Alongside this, sub-optimal access to computers and internet connectivity whenever needed was also prevalent when it came to students' needs. These factors affected the stress of students’ e-learning exposure. Bangladeshi academia was always entirely focused on the in-person educational structure where students were never habituated with technological dependency. With the outbreak of COVID-19, most students were compelled to shift online learning structure to avoid their study gap. This sudden shift could not prepare the students equally from gender, rurality, and non-centralized perspectives. Therefore, females being less exposed to the internet and computer systems, specifically when it comes to rural areas, are often disadvantaged. The students in the periphery do not get computer and internet connections available whenever needed. This could be a plausible explanation for being significantly associated with stress. Several studies were found in agreement with our findings ( Barrot *et al.*, 2021; Keskin & Yurdugül, 2020; Purnama *et al.*, 2021). On the other hand, Purnama *et al*. (2021), assessed the risk of internet misusing and cyberbullying increasing along with the free access of computers and the internet.

Like availability, the usability of technology was also observed to be significantly sub-optimum among females, rural and students outside Dhaka. It was found to affect the stress score of students significantly. Numerous studies found students and teachers were struggling when it came to self-efficacy of internet use. Students have seen issues due to their lack of knowledge on internet usage in an academic context and teachers' struggling due to lack of internet exposure ( Bao, 2020; Churiyah *et al.*, 2020; Siron *et al.*, 2020). From a general perspective, people in Bangladesh are used to internet usage, but mainly for social media purposes. When stratified by gender, Bangladeshi females were found less technological usability, which can explain that they are primarily occupied doing chores. A similar scenario was found outside of Dhaka, where students may optimize their time with outdoor activities. In rural settings, students maintain routine lifestyle that is more scheduled with in-person interactions. These might be reasons beneath the observed prevalence, hence, affecting students’ stress levels.

Female, rural, and outside Dhaka were seen to have sub-optimum prevalence when communicating via e-mail. This factor not only was prevalent but also accelerated the stress among the students. E-mail communication is not a common way of interacting for this generation in Bangladesh. Social networking systems have changed their communication habits and made them more informal. Bangladeshi students are more comfortable making a phone call or sending a text in or messaging on Facebook, WhatsApp etc. However, regarding the rural perspective, students and teachers prefer to respond via mobile call or direct communications rather than e-mail. Moreover, Bangladeshi females and peripheric students are less technologically adept due to the behavioral and cultural norms practiced for a long time. Studies supporting our findings showed the importance of online communication in this digital era ( Alghamdi *et al.*, 2020; Walker *et al.*, 2016).

A significant difference in sub-optimal means was found with office software usability compared to the optimal usability. Many Turkish, Indonesian, and Jordanian studies explored that expertise and skills in office software usage could benefit students of all levels ( Çevik & Bakioğlu, 2021; Siron *et al.*, 2020). From an overall perspective, students in our country are less used to the usability of technology such as Microsoft office. The prominent structure of regular face-to-face classes never required them to be. Thus, the complete curriculum shift depending on software usage could be the reason for such findings, causing extreme stress levels in the students.

In this study, instant messaging, file hosting services, learning management systems, and online forum usability were some of the signs found as associated factors. These affected the students’ stress levels with a higher difference of means compared to optimality. The reason could be the never-needed functionality for rural students in online forms of communication. As for the female and non-centralized students, women here are less keen to explore the numerous technological mediums and devices except for the most common platforms. Studies from various continents found similar outcomes and concluded with its importance in developing a solid e-learning system ( Purnama *et al.*, 2021). Here in Bangladesh, another general reason could be the expense regarding the usage, looking at most university students' cultural and socioeconomic backgrounds.

## 5. Limitations and strengths

Due to lockdown, the online snowball sampling technique was applied that is one of the limitations of our study, compromising the randomization of students. Recall bias of the respondent could not be excluded due to the self-reported online questionnaire approach. A tool to collect qualitative and quantitative data could bring up some narratives of the students’ experiences for a deeper understanding.

This study enrolled students with a moderate sample size to be addressed as a representative study keeping in mind the lockdown situation of university students. This study also considered the various factors associated with the technological barriers that the e-learning community is facing. It may help policymakers developing the technological ground for academia to avoid educational hindrances due to any emerging situation, similar to the COVID-19 pandemic. For further research recommendation, a more rigorous analytical study on the subject matter could be informative.

## 6. Conclusion

This study assessed the technological stands of the university students' usability and technical support availability. The study found that lack of technological stands affects the stress level of the students enrolled in an online curriculum. However, the findings strongly suggest the relevant authorities take steps to revitalize the curriculum of e-learning to be adaptive to technology. Such awareness steps would facilitate the students' technical knowledge development, which is essential for building digital Bangladesh and accelerating digital literacy. Hence, preparing the academicians always to be more ready to tackle hurdles such as pandemics and transform the curriculum into e-learning from traditional rapidly whenever needed.

## 7. Recommendations

Assessing the prevalence of sub-optimum and optimum technological availability and usability among university students and their association with perceived e-learning stress may highlight the education system's infrastructure. Insights from identifying the effect of students’ technological condition and supporting initiatives to reduce their stress level can be used to derive the corrective action(s) that needs to be assessed for building digital Bangladesh.

## Data availability

### Underlying data

Zenodo: Availability and use of technology for e-learning in Bangladesh https://doi.org/10.5281/zenodo.5748586.

This project contains the following underlying data:

• Data.xls (raw data from questionnaires)

Data are available under the terms of the Creative Commons Attribution 4.0 International license (CC-BY 4.0).

## Consent

Informed electronic consent was obtained for all the participants involved in the study.
