# Supplementary material for: Availability and use of technology for e-learning: to what extent do these impact Bangladeshi university students? A cross-sectional study
Source: F1000Res. 2021 Dec 15;10:1285. [Version 1] doi: 10.12688/f1000research.75532.1 (PMC9020528; doi:10.12688/f1000research.75532.1)
Supplement: Peer review history

# Peer review: Reviewer response for version 1

**DOI:** 10.5256/f1000research.79415.r134980

**Published:** 2022-04-20

**Authors:** Shaher Hamaideh

**Affiliations:** 1 Community and Mental Health Nursing Department, Faculty of Nursing, The Hashemite University, Zarqa, Jordan

The paper addressed an important issue about the availability and use of technology for e-learning among university students. This has become an important issue globally due to the pandemic. The authors present the topic appropriately with a good flow, and used appropriate and adequate references and citations. Most of the references are relatively up to date and specifically address the issue of e-learning among university students especially in the period of COVID-19. However, the introduction is a little bit long. The authors may shorten it and combine similar points where applicable. 

 Using a cross-sectional design to collect data online (using Google forms) is appropriate for the purpose of this study. The data was analyzed and interpreted in the text appropriately. However, the tables are too crowded which makes it difficult for the readers to follow. The conclusions drawn from the study were adequately supported by the results.

Is the work clearly and accurately presented and does it cite the current literature?

Yes

If applicable, is the statistical analysis and its interpretation appropriate?

Yes

Are all the source data underlying the results available to ensure full reproducibility?

Yes

Is the study design appropriate and is the work technically sound?

Yes

Are the conclusions drawn adequately supported by the results?

Yes

Are sufficient details of methods and analysis provided to allow replication by others?

Yes

Reviewer Expertise:

Mental health nursing

I confirm that I have read this submission and believe that I have an appropriate level of expertise to confirm that it is of an acceptable scientific standard.

# Peer review: Reviewer response for version 1

**DOI:** 10.5256/f1000research.79415.r129294

**Published:** 2022-04-19

**Authors:** Enric Serradell-López

**Affiliations:** 1 Economics and Business Department, Universitat Oberta de Catalunya, Barcelona, Spain

The research developed by the authors is focused on one of the hot topics of how the Covid-19 pandemic affects students. They propose the concept of perceived e-learning stress as an attempt to measure the influence of the pandemic on students. As all the indicators are based on perceptions, it is not easy to generalize, but the conclusions obtained are interesting due to the fact that more than one thousand students answered the questionnaire. The data collection is also interesting as they used social networks.

 The statistical analysis and its interpretation is correct; however, more statistical analysis could be developed, for example cluster or factorial analysis. The underlying data is available so I'd like to congratulate the authors for that. The conclusions are correct and follow the logical of the article. When we talk about e-learning it should be normal to detect the technology needs of the students. The recommendations should be followed by policymakers who must provide sufficient resources to public universities.

Is the work clearly and accurately presented and does it cite the current literature?

Yes

If applicable, is the statistical analysis and its interpretation appropriate?

Yes

Are all the source data underlying the results available to ensure full reproducibility?

Yes

Is the study design appropriate and is the work technically sound?

Yes

Are the conclusions drawn adequately supported by the results?

Yes

Are sufficient details of methods and analysis provided to allow replication by others?

Yes

Reviewer Expertise:

E-learning, Management, Business games, Strategy

I confirm that I have read this submission and believe that I have an appropriate level of expertise to confirm that it is of an acceptable scientific standard.

# Peer review: Reviewer response for version 1

**DOI:** 10.5256/f1000research.79415.r124018

**Published:** 2022-03-15

**Authors:** Saeed Anwar

**Affiliations:** 1 Prime Institute of Public Health, Riphah International University, Islamabad, Pakistan

The research by the authors is an important inquiry into the crucial issue of e-learning imposed by the new normal and the associated challenges in an LMIC (quite under-researched in these contexts). 

 The authors have planned, managed and written the research very well. Source data is available for almost all the variables of the study. Descriptive and inferential statistics have been applied and related appreciably. The limitations and strengths have been narrated reasonably well, though they might have been even better communicated in user friendly terms by disaggregating them under separate subtitles of ‘LIMITATIONS’ and ‘STRENGTHS’, rather than binding them under one subtitle.

Is the work clearly and accurately presented and does it cite the current literature?

Yes

If applicable, is the statistical analysis and its interpretation appropriate?

Yes

Are all the source data underlying the results available to ensure full reproducibility?

No source data required

Is the study design appropriate and is the work technically sound?

Yes

Are the conclusions drawn adequately supported by the results?

Yes

Are sufficient details of methods and analysis provided to allow replication by others?

Yes

Reviewer Expertise:

NA

I confirm that I have read this submission and believe that I have an appropriate level of expertise to confirm that it is of an acceptable scientific standard.
